# Prior Adjuvant Tamoxifen Treatment in Breast Cancer Is Linked to Increased AIB1 and HER2 Expression in Metachronous Contralateral Breast Cancer

**DOI:** 10.1371/journal.pone.0150977

**Published:** 2016-03-09

**Authors:** Sara Alkner, Pär-Ola Bendahl, Anna Ehinger, Kristina Lövgren, Lisa Rydén, Mårten Fernö

**Affiliations:** 1 Division of Oncology and Pathology, Department of Clinical Sciences, Lund University, Medicon Village, SE-223 63, Lund, Sweden; 2 Skåne Clinic of Oncology, Skåne University Hospital Lund, SE-222 41, Lund, Sweden; 3 Department of Pathology and Cytology, Blekinge County Hospital, SE-371 85, Karlskrona, Sweden; 4 Clinic of Surgery, Skåne University Hospital Lund, SE-222 41, Lund, Sweden; University of North Carolina School of Medicine, UNITED STATES

## Abstract

**Aim:**

The estrogen receptor coactivator Amplified in Breast Cancer 1 (AIB1) has been associated with an improved response to adjuvant tamoxifen in breast cancer, but also with endocrine treatment resistance. We hereby use metachronous contralateral breast cancer (CBC) developed despite prior adjuvant tamoxifen for the first tumor as an *“in vivo”*-model for tamoxifen resistance. AIB1-expression in the presumable resistant (CBC after prior tamoxifen) and naïve setting (CBC without prior tamoxifen) is compared and correlated to prognosis after CBC.

**Methods:**

From a well-defined population-based cohort of CBC-patients we have constructed a unique tissue-microarray including >700 patients.

**Results:**

CBC developed after adjuvant tamoxifen more often had a HER2-positive/triple negative-subtype and a high AIB1-expression (37% *vs*. 23%, p = 0.009), than if no prior endocrine treatment had been administered. In patients with an estrogen receptor (ER) positive CBC, a high AIB1-expression correlated to an inferior prognosis. However, these patients seemed to respond to tamoxifen, but only if endocrine therapy had not been administered for BC1.

**Conclusions:**

Metachronous CBC developed after prior endocrine treatment has a decreased ER-expression and an increased HER2-expression. This is consistent with endocrine treatment escape mechanisms previously suggested, and indicates metachronous CBC to be a putative model for studies of treatment resistance *“in vivo”*. The increased AIB1-expression in CBC developed after prior tamoxifen suggests a role of AIB1 in endocrine treatment resistance. In addition, we found indications that the response to tamoxifen in CBC with a high AIB1-expression seem to differ depending on previous exposure to this drug. A different function for AIB1 in the tamoxifen treatment naïve vs. resistant setting is suggested, and may explain previously conflicting results where a high AIB1-expression has been correlated to both a good response to adjuvant tamoxifen and tamoxifen resistance.

## Introduction

Adjuvant tamoxifen improves survival after estrogen receptor (ER) positive breast cancer. However, recurrences still occur, and in the metastatic setting patients eventually develop resistance. An interesting biomarker in relation to endocrine therapy is the ER-coactivator “Amplified in Breast Cancer 1” (AIB1) [1–5]. Overexpression of AIB1 is present in 30–60% of human breast cancers and correlates with an aggressive tumor phenotype (HER2-overexpression, DNA-nondiploidy, high histological grade, and high Ki67/S-phase fraction) [1–3, 6–12]. We have, in both a randomized tamoxifen-trial including 349 premenopausal women and in independent cohorts, investigated AIB1’s prognostic and treatment predictive value for tamoxifen [13, 14]. We found patients with AIB1-high tumors to respond very well to tamoxifen. Patients with low AIB1, on the other hand, had a better prognosis from the beginning but this was not further improved by tamoxifen. Without tamoxifen, a high AIB1 was consistently a negative prognostic factor. These results have been confirmed in another randomized tamoxifen-trial including 910 postmenopausal women [15].

Hence, a high AIB1-expression seems to predict tamoxifen response. However, a deregulation of coactivators including AIB1 has also been suggested to be of importance for tamoxifen resistance. A few studies have shown increased AIB1 in tamoxifen-resistant cell lines [16–18], while others have found cell lines with increased ER/AIB1-expression to respond well to tamoxifen [19, 20]. A high AIB1 has also been associated with a worse disease-free survival in unselected tamoxifen treated cohorts [7, 9, 21]. These conflicting results could indicate a different role of AIB1 in the treatment naïve *vs*. the resistant setting. Possible explanations for this are considered in the “discussion section” below.

A new model to study endocrine treatment escape mechanisms *“in vivo”* is metachronous contralateral breast cancer (CBC) developed despite adjuvant treatment given for the first tumor (BC1), and hence presumably resistant to this treatment. Indeed, CBC developed after prior endocrine therapy is to a larger extent ER-negative, and prior endocrine therapy, chemotherapy and radiotherapy have all been associated with a worse prognosis once diagnosed with CBC [22–25]. From a well-defined population-based cohort of CBC-patients we have constructed a unique tissue-microarray (TMA) including >700 patients with metachronous CBC. For each patient, detailed epidemiological, tumor and treatment information is available.

We now use this unique material to investigate AIB1-tumor-expression in the treatment naïve (CBC without prior tamoxifen) *vs*. the presumably resistant setting (CBC despite prior tamoxifen), and correlate this to prognosis after CBC. Considering our previous studies we expect AIB1 to be a negative prognostic factor in patients not given tamoxifen for an ER-positive CBC. However, ER-positive CBC with high AIB1 should respond well to tamoxifen if not previously exposed to this drug. On the other hand, increased AIB1-expression after previous tamoxifen may instead represent an endocrine treatment escape mechanism, and these tumors would then presumably be resistant to tamoxifen.

Since inhibitors of AIB1 are currently being explored as a possibly future breast cancer treatment [26], we find it important to further investigate the function of AIB1 in relation to different tumor characteristics and treatment settings, and we think this study contributes to that knowledge.

## Patients and Methods

### Tissue microarray

**Fig 1:**
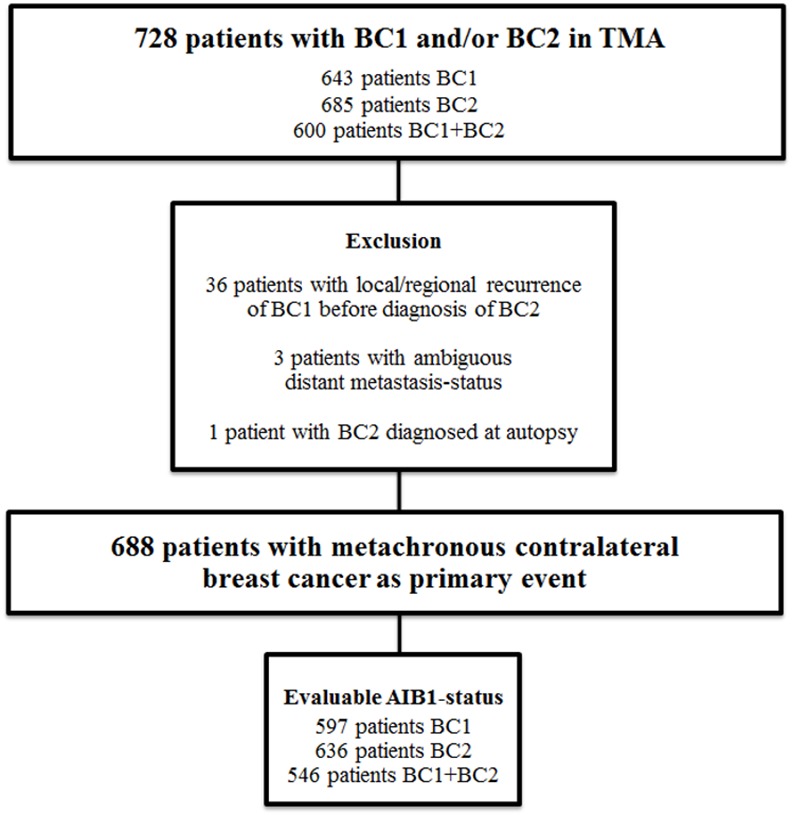
Flow-chart of inclusion *vs*. exclusion in the study cohort. In analysis 36 patients with a local/regional recurrence of BC1 before diagnosis of BC2 were excluded in order not to confuse the results by eventual treatment given for the recurrence. We also excluded 3 patients with ambiguous distant metastasis-status and 1 patient with BC2 diagnosed at autopsy. Abbreviations: AIB1 *Amplified in breast cancer 1*, BC1 f*irst breast cancer*, BC2 *second breast cancer*, TMA *tissue microarray*

Inclusion criteria, data abstraction, and TMA-construction have been described before [23]. Briefly, all patients within the Southern Swedish Healthcare Region with two breast cancers in the Swedish Cancer Registry (BC2 diagnosed 1977–2007) were included. Clinical data were abstracted from individual charts and paraffin-embedded tissue collected. We focused on metachronous CBC (≥3 months between tumors), excluding patients with synchronous CBC, distant metastasis or another malignancy diagnosed before BC2, or with BC2 found only in the axilla. For the remaining 764 patients, paraffin-blocks were available for 643 BC1 and 685 BC2 (both tumors in 600 cases), giving a total of 728 patients included in the TMA (Fig 1). Prognosis and hormone receptor status in relation to both tumors and treatment has been presented before [25]. Since all patients with another malignancy before their CBC were excluded, no patients had received chemotherapy or endocrine therapy prior to diagnosis of their first breast cancer. However, 6 patients had received previous radiotherapy towards the chest due to benign afflictions. Neoadjuvant treatment was given to 5 of the 62 patients that received chemotherapy for BC1, and to 2 of the 47 patients that received chemotherapy for BC2. Patient and tumor characteristic in relation to AIB1 are described in Table 1.

**Table 1:** Patient and tumor characteristics in relation to AIB1.

N = 688		First breast cancer, N (%)			P-value^1^		Second breast cancer, N (%)			P-value^1^
AIB1 missing for 91 BC1, 52 BC2	N	AIB1 Low	AIB1 Medium	AIB1 High		N	AIB1 Low	AIB1 Medium	AIB1 High	
		N = 212 (35%)	N = 290 (49%)	N = 95 (16%)			N = 171 (27%)	N = 301 (47%)	N = 164 (26%)	
**Date of diagnosis**					0.004					0.002
<1977	91	42 (46)	33 (36)	16 (18)		0	0	0	0	
1977–1986	181	74 (41)	83 (46)	24 (13)		120	44 (37)	49 (41)	27 (23)	
1987–1996	239	77 (32)	123 (51)	39 (16)		219	66 (30)	101 (46)	52 (24)	
1997–2007	86	19 (22)	51 (59)	16 (19)		297	61 (21)	151 (51)	85 (29)	
**Age at diagnosis**										0.5
<50 years	173	73 (42)	71 (41)	29 (17)	0.2	72	15 (21)	39 (54)	18 (25)	
≥50 years	424	139 (33)	219 (52)	66 (16)		564	156 (28)	262 (46)	146 (26)	
**Node status**										0.01
N0	374	129 (34)	190 (51)	55 (15)	0.5	339	95 (28)	166 (49)	78 (23)	
N+	186	66 (35)	83 (45)	37 (20)		190	44 (23)	80 (42)	66 (34)	
Number if N+ Median (range)		2 (1–15)	3 (1–33)*	2.5 (1–12)			2.5 (1–19)	3 (1–23)	3 (1–21)	
*Missing*	*37*	*17*	*17*	*3*		*107*	*32*	*55*	*20*	
**Size**										0.04
≤20 mm	355	120 (34)	180 (51)	55 (15)	0.7	444	120 (27)	225 (51)	99 (22)	
>20 mm	203	75 (37)	95 (47)	33 (16)		174	45 (26)	69 (40)	60 (34)	
Median (range)*		18 (1–80)*	17 (1–100)*	18.5 (1–70)*			15 (1–80)*	14.5 (1–110)*	18 (1–75)*	
*Missing*	*39*	*17*	*15*	*7*		*18*	*6*	*7*	*5*	
**Stage**										0.003
I	268	89 (33)	140 (52)	39 (15)	0.4	273	78 (29)	141 (52)	54 (20)	
II	185	69 (37)	88 (48)	28 (15)		158	38 (24)	68 (43)	52 (33)	
III	63	19 (30)	29 (46)	15 (24)		83	19 (23)	33 (40)	31 (37)	
*Missing*	*81*	*35*	*33*	*13*		*122*	*36*	*59*	*27*	
**ER-status**										<0.001
<10	99	39 (39)	36 (36)	24 (24)	0.6	105	21 (20)	34 (32)	50 (48)	
≥10	490	167 (34)	252 (51)	71 (14)		520	144 (28)	262 (50)	114 (22)	
*Missing*	*8*	*6*	*2*	*0*		*11*	*6*	*5*	*0*	
**PR-status**										<0.001
<10	165	55 (33)	68 (41)	42 (25)	0.02	211	43 (20)	85 (40)	83 (39)	
≥10	424	151 (36)	220 (52)	53 (13)		409	122 (30)	208 (51)	79 (19)	
*Missing*	*8*	*6*	*2*	*0*		*16*	*6*	*8*	*2*	
**HER2-status**										<0.001
Negative (0 to 2+)	541	199 (37)	265 (49)	77 (14)	<0.001	580	161 (28)	285 (49)	134 (23)	
Positive (3+)	40	7 (18)	17 (43)	16 (40)		37	2 (5)	8 (22)	27 (73)	
*Missing*	*16*	*6*	*8*	*2*		*19*	*8*	*8*	*3*	
**Ki67**										<0.001
≤20	476	187 (39)	231(49)	58 (12)	<0.001	493	151 (31)	245 (50)	97 (20)	
>20	104	18 (17)	51 (49)	35 (34)		118	9 (8)	46 (39)	63 (53)	
*Missing*	*17*	*7*	*8*	*2*		*25*	*11*	*10*	*4*	
**Subtype**										<0.001
Luminal A-like	355	135 (38)	183 (52)	37 (10)	<0.001	335	109 (33)	181 (54)	45 (13)	
Luminal B-like HER2-	112	29 (26)	56 (50)	27 (24)		152	28 (18)	72 (47)	52 (34)	
Luminal B-like HER2+	17	2 (12)	9 (53)	6 (35)		19	1 (5)	4 (21)	14 (74)	
HER2+	21	4 (19)	7 (33)	10 (48)		16	1 (6)	3 (19)	12 (75)	
Triple Negative	63	26 (41)	24 (38)	13 (21)		75	15 (20)	27 (36)	33 (44)	
*Missing*	*29*	*16*	*11*	*2*		*39*	*17*	*14*	*8*	
**Time-interval between tumors**										0.9
<5 year	268	88 (33)	141 (53)	39 (15)	0.7	271	70 (26)	133 (49)	68 (25)	
≥5 years	329	124 (38)	149 (45)	56 (17)		365	101 (28)	168 (46)	96 (26)	
**Prior radiotherapy**										0.7
No						242	67 (28)	115 (48)	60 (25)	
Yes						389	103 (26)	185 (48)	101 (26)	
*Missing*						*5*	*1*	*1*	*3*	
**Prior chemotherapy**										0.01^b^
No						568	162 (29)	266 (47)	140 (25)	
Combination^a^						53	7 (13)	30 (57)	16 (30)	
Cyclophosphamide only						9	1 (10)	3 (33)	5 (56)	
*Missing*						*6*	*1*	*2*	*3*	
**Prior endocrine treatment**										0.009^d^
No						483	136 (28)	238 (49)	109 (23)	
Tamoxifen						130	31 (24)	51 (39)	48 (37)	
Other^c^						17	3	10	4	
*Missing*						*6*	*1*	*2*	*3*	

**Abbreviations: BC1** *first breast cancer* **BC2** *second breast cancer*, **ER** *estrogen receptor*, **N+** *lymph-node metastases*, **N0** *no lymph-node metastases*, **N** *number*, **node status** *lymph-node status*, **PR** *progesterone receptor*.

^a^ Combination therapy. Most common regimes: FEC (fluorouracil, epirubicin, cyclophosphamide), CMF (cyclophosphamide, methotrexate and fluorouracil) or anthracyclins combined with taxanes. One patient with weekly doxorubicin included in this group

^b^ Chemotherapy *vs*. no chemotherapy.

^c^ Patients with other endocrine treatment than tamoxifen were excluded from analyses involving effect of tamoxifen treatment for BC1 and/or BC2. In 13 patients the endocrine treatment used was oophorectomy, 1 patient received oophorectomy + tamoxifen, 1 patient tamoxifen followed by an aromatase inhibitor, and 2 patients androgens.

^d^ Prior tamoxifen *vs*. no prior endocrine treatment

^1^ χ^2^-test for trend except for subtype where a regular chi2-test was used due to unordered categories

The project was approved by the Regional Ethical Review Board of Lund University (LU240-01) and carried out in accordance with the code of ethics of the World Medical Association. Since the study handles saved paraffin material, often several decades old, informed consent was not possible to retrieve from all patients. However, all data was analyzed and presented anonymously. In addition a note was published in the local paper, informing all previous breast cancer patients about the possibility to contact the research group if they did not want their tumor tissue to be used in scientific studies. This procedure was accepted by the Regional Ethical Review Board.

### Immunohistochemistry

Immunohistochemistry (IHC) was performed in an Autostainer-*Plus*, Dako (Ki67 M7240-Dako, ER RM-9101 ThermoScientific, progesterone receptor (PR) M3569-Dako) as previously descried [14]. For HER2 the Ventana Benchmark system was used (Ventana 790–2991). ER, PR, HER2, and Ki67 were reevaluated by a pathologist (AE). In line with Swedish clinical standard during this period tumors with ≥10% stained nuclei were considered ER-/PR-positive. HER2 3+ was considered HER2-positive, and Ki67-expression in >20% of cell nuclei considered Ki67-high.

As primary antibody for AIB1 detection a mouse-monoclonal IgG antibody was used at 1:100 dilution (Cat no #611105 BD Bioscience, CA, USA), as previous described [7, 14]. This antibody has been used in several previous clinical trials [2, 7, 8], and its specificity has been confirmed by Western blot, Northern blot, and *in situ* hybridization [2, 8, 27]. IHC-staining (nuclear) was examined by two independent viewers blinded for clinical/tumor-characteristics (SA, KL). Each sample was semi-quantitative scored from 0–3 for percentage of stained nuclei and staining intensity. Proportion score 0 represented no stained nuclei, 1: 1%–10%, 2: 11%–50%, and 3: 51%–100%. Staining intensity 0 represented negative staining, 1 weak, 2 moderate, and 3 intense staining [7, 13, 14]. Proportion and intensity scores were added to a total score ranging from 0 to 6. When scores differed more than one step between viewers (6%) cases were reexamined to reach consensus, otherwise the mean score was used. In case of discrepant staining between the two cores from the same patient, the highest score was used. Surrogate definitions of intrinsic subtypes were defined using IHC-annotated biomarker according to the St Gallen-guidelines [28].

### Statistical analysis

Survival-data and cause of death was retrieved from the Swedish National Board of Health and Welfare (March 2014), and breast cancer mortality (BCM) chosen as primary end-point. BCM was defined as breast cancer death or death after metastasis. Event-free survival was measured from CBC-diagnosis.

For statistical calculations, the software package Stata 11.2 (StataCorp, USA) was used. Associations between AIB1-values/prior tamoxifen and patient/tumor-characteristics were evaluated with the χ^2^-test or the χ^2^-test for trend. Prognosis after BC2 was summarized graphically as cumulative BCM and cause-specific Cox-regression, treating competing events as censoring, was used to estimate hazard ratios (HR). Assumptions of proportional hazards were checked graphically. Logistic regression was used to determine risk factors for developing a CBC with certain features. 95% confidence intervals (CI), corresponding to a p-value threshold of 0.05, were used to summarize variability in estimated effects.

Approximately 90% of patients with endocrine therapy for BC1 received tamoxifen (Table 1). Patients with other endocrine treatment than tamoxifen for BC1 were excluded from analyses regarding tamoxifen. Other prior adjuvant treatment did not significantly differ between patients with *vs*. without tamoxifen for BC1 (radiotherapy 61% *vs*. 63%, chemotherapy 6% *vs*. 11%). All analyses were repeated for patients receiving only tamoxifen as adjuvant treatment for BC1 *vs*. patients without any prior adjuvant treatment with similar results.

Multivariable analyses were adjusted for characteristics and treatment of both tumors (calendar period of diagnosis, age, time-interval to BC2, TNM-stage, ER, AIB1, HER2, Ki67, endocrine treatment, chemotherapy and radiotherapy). Regarding calendar period, the material was divided in thirds by diagnosis-date of BC1.

## Results

### AIB1 expression and cut-off

We have previously used a cut-off of ≥5 to define high *vs*. low AIB1, which in previous cohorts has identified roughly 50% of tumors as AIB1-high. However, in this cohort several tumors had medium-high scores (5 and 5.5), giving us 65% of BC1 and 73% of BC2 with AIB1-scores ≥5. In addition, analyses below showed tumors with medium-scores to often express features in-between those with low and high AIB1. Due to this, and in order to make our analysis more comparable to previous studies, where often only the highest quartile of AIB1-expression was regarded “high AIB1” [8, 9], we chose to analyze AIB1-expression in three stages–low (score <5), medium (score 5 or 5.5) and high (score 6).

### AIB1 in relation to patient and tumor characteristics

In both BC1 and BC2 a high tumor AIB1-expression was associated with ER- and/or PR-negativity, HER2-overexpression, and high Ki67 (Table 1). The AIB1-level of BC1 also correlated to that of BC2 (χ^2^-test for trend, p = 0.004). In BC2 a high AIB1 correlated to a high TNM-stage. Higher AIB1-levels were also observed with a later calendar-period of diagnosis.

### Effect of adjuvant treatment on AIB1-, HER2- and Ki67-expression in metachronous CBC

CBC developed after prior tamoxifen more often had a high AIB1- and/or HER2-expression than if no prior tamoxifen had been given (Table 2), and especially so in ER-negative CBC. CBC after prior tamoxifen was also more often of the HER2-postive or triple negative subtype. Ki67 in BC2 did not differ in relation to prior tamoxifen in the whole cohort, although significantly lower levels were observed in ER-negative CBC after prior tamoxifen. Correlations between prior tamoxifen and biomarker-expression in BC2 were even stronger when considering only CBC diagnosed within a short time-interval from BC1 (<5 years) (data not shown).

**Table 2:** Biomarkers in BC2 in relation to prior tamoxifen.

N = 663^a^	All CBC		P-value^1^	ER-positive CBC		P-value^1^	ER-negative CBC		P-value^1^
ER-status missing for 57 CBC	No prior tam	Prior tam		No prior tam	Prior tam		No prior tam	Prior tam	
	N = 522 (79%)	N = 141 (21%)		N = 412 (81%)	N = 95 (19%)		N = 66 (67%)	N = 33 (33%)	
**AIB1**									0.04
Low	136 (28)	31 (24)	0.009	119 (29)	23 (25)	0.1	14 (21)	5 (15)	
Medium	238 (49)	51 (39)		208 (51)	44 (47)		26 (39)	6 (18)	
High	109 (23)	48 (37)		83 (20)	26 (28)		26 (39)	22 (67)	
*Missing*	*39*	*11*		*2*	*2*		*0*	*0*	
**HER2**	* *								0.07
Negative	449 (95)	114 (89)	0.008	391 (97)	90 (95)	0.4	56 (88)	24 (73)	
Positive	22 (5)	14 (11)		14 (3)	5 (5)		8 (13)	9 (27)	
*Missing*	*51*	*13*		*7*	*0*		*2*	*0*	
**Ki67**									0.01
≤20%	379 (81)	101 (80)	0.8	352 (88)	79 (85)	0.5	25 (40)	22 (67)	
>20%	88 (19)	25 (20)		50 (12)	14 (15)		38 (60)	11 (33)	
*Missing*	*55*	*15*		*10*	*2*		*3*	*0*	
**Subtype**			0.005						
Luminal A-like	266 (58)	59 (48)							
Luminal B-like HER2-	120 (26)	29 (24)							
Luminal B-like HER2+	14 (3)	5 (4)							
HER2+	7 (2)	8 (7)							
Triple Negative	50 (11)	21 (17)							
*Missing*	*65*	*19*							

**Abbreviations: AIB1** *amplified in breast cancer 1*, **CBC** *contralateral breast cancer*, **ER** *estrogen receptor*, **HER2** *human epidermal growth factor receptor 2*, **Info** *information*, **N** *number*, **PR** *progesterone receptor*, **tam** *tamoxifen*.

^a^ Patients with prior tamoxifen or no prior endocrine therapy included in analysis.

^1^ χ^2^-test except for AIB1 where a χ^2^-test for trend was used due to several ordered categories.

No significant change in percentage of CBC with high AIB1, HER2 or Ki67 was observed when comparing patients with only prior radiotherapy (N = 275) *vs*. no prior adjuvant treatment (N = 190). Patients with only prior chemotherapy were too few to analyze alone (N = 11). However, in patients receiving chemotherapy +/- radiotherapy (N = 57, patients with endocrine therapy excluded), prior chemotherapy correlated to a high CBC AIB1-expression (31% *vs*. 22%, p = 0.03) and in ER-positive CBC also to HER2-overexpression (10% *vs*. 3%, p = 0.02). No difference was seen for Ki67.

A multivariable logistic regression analysis adjusted as described above showed only prior tamoxifen to be an independent risk factor for developing a CBC with high AIB1-expression (OR 2.0, 95%CI 1.1–3.5, p = 0.02).

### AIB1-expression in relation to prognosis after development of CBC

**Fig 2:**
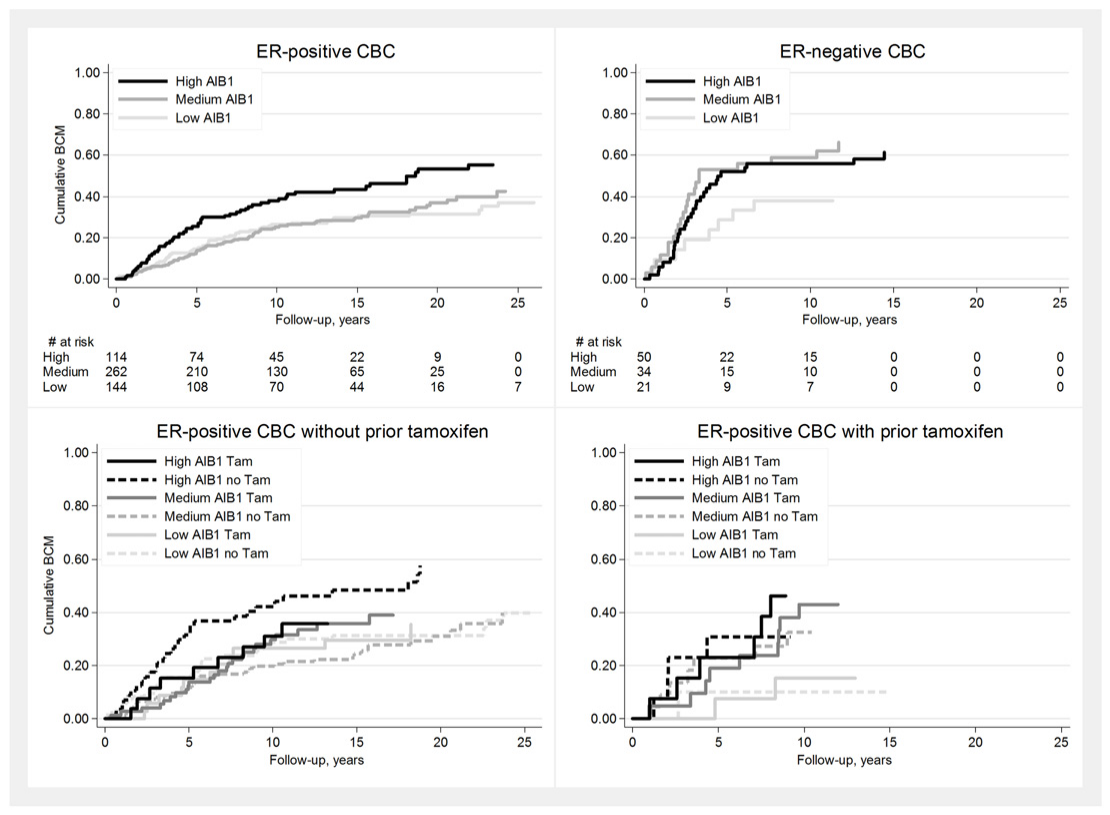
**Breast cancer mortality in relation to AIB1-expression in the CBC** A. ER-positive CBC. B. ER-negative CBC. C. ER-positive CBC not previously exposed to tamoxifen. D. ER-positive CBC developed despite prior tamoxifen. A high AIB1-expression was associated with a worse BCM in ER-positive CBC (A), but not in ER-negative CBC (B). However, in ER-positive CBC not previously exposed to tamoxifen, a worse BCM with a high-AIB1 was only seen if tamoxifen was not given for the CBC (C). If treated with tamoxifen, on the other hand, AIB1-high CBC had an equally good prognosis as tumors with lower AIB1-expression. This is consistent with previous studies, and indicates a response to tamoxifen in AIB1-high tumors. Conversely, no difference in prognosis was seen in relation to tamoxifen treatment in ER-positive AIB1-high CBC developed despite prior tamoxifen (D). This could reflect an unresponsiveness and possible resistance to tamoxifen in these tumors. All plots were curtailed when <5 individuals remained at risk.

A high AIB1-expression was associated with a worse prognosis in ER-positive CBC (Fig 2A and 2B, Table 3). Although there seemed to be a trend for a worse survival with a high AIB1 also in ER-negative CBC, this did not reach statistical significance. This may be due to the smaller number of patients in this subgroup. AIB1 in BC1 did not affect prognosis after diagnosis of BC2.

**Table 3:** Prognosis after CBC in relation to AIB1-status of BC2.

	Univariable						Multivariable^1^					
AIB1 Low Reference	AIB1 Medium			AIB1 High			AIB1 Medium			AIB1 High		
	HR	95%CI	p-value	HR	95%CI	p-value	HR	95%CI	p-value	HR	95%CI	p-value
**All patients (N = 636)**^a^	1.1	0.8–1.5	0.5	1.9	1.4–2.7	<0.001	1.4	0.8–2.3	0.2	1.8	1.0–3.1	0.05
**ER-positive CBC (N = 520)**	1.0	0.7–1.5	0.9	1.8	1.2–2.6	0.004	1.5	0.9–2.6	0.1	2.5	1.3–4.9	0.006
*Prior tamoxifen (N = 93)*	*2*.*5*	*0*.*9–6*.*8*	*0*.*08*	*3*.*7*	*1*.*3–11*	*0*.*02*	*15*	*1*.*3–173*	*0*.*03*	*5*.*4*	*0*.*7–43*	*0*.*1*
*No prior tamoxifen (N = 410)*	*0*.*9*	*0*.*6–1*.*3*	*0*.*6*	*1*.*6*	*1*.*0–2*.*5*	*0*.*03*	*1*.*4*	*0*.*8–2*.*6*	*0*.*3*	*2*.*2*	*1*.*0–4*.*8*	*0*.*04*
**ER-negative CBC (N = 105)**	1.7	0.79–3.5	0.2	1.3	0.66–2.7	0.4	3.3	0.65–17	0.2	1.2	0.28–5.3	0.8

^a^ ER-status missing for 11 patients. Patients with prior tamoxifen or no prior endocrine therapy included in analysis regarding endocrine treatment.

^1^ Multivariable analyses adjusted for calendar-period of diagnosis, time-interval between tumors, age, and characteristics and treatment given for BC1 and BC2 (TNM-stage, ER, AIB1, HER2, Ki67, radiotherapy, chemotherapy, and tamoxifen). Subgroup analyses regarding ER-status and tamoxifen of course not adjusted for these variables.

**Abbreviations: AIB1** *amplified in breast cancer 1*, **CBC** *contralateral breast cancer*, **ER** *estrogen receptor*, **N** *number*.

A high AIB1-expression correlated to a worse prognosis in ER-positive CBC regardless of prior tamoxifen treatment. However, in patients not previously exposed to tamoxifen a high-AIB1 was only associated with a higher BCM if tamoxifen was not given for BC2 (Multivariable analysis: HR 2.5, p = 0.04, 95%CI 1.1–6.1). While patients with high AIB1-tumors receiving tamoxifen for their CBC, had an equally good prognosis as those with lower AIB1-levels (Fig 2C). In patients developing ER-positive CBC despite prior tamoxifen, on the other hand, high AIB1 was linked to a higher BCM regardless if tamoxifen was given for the CBC or not (Fig 2D). However, this did not reach significance in these smaller subgroups. Results were repeated for patients developing CBC within 5 years of BC1 with similar results, although these patients were too few for reliable subgroup analyses (data not shown).

## Discussion

CBC diagnosed after prior adjuvant treatment is presumably resistant, and could hence be used as an *“in vivo”*-model for studies of adjuvant treatment resistance. Using a unique TMA including >700 CBC-patients we hereby investigate if CBC AIB1-expression is increased after previous tamoxifen treatment, possibly reflecting a treatment escape mechanism. We also evaluate AIB1-expression in relation to prognosis and tamoxifen response after diagnosis of CBC, both in the treatment naïve (CBC without prior tamoxifen) and presumably resistant setting (CBC despite prior tamoxifen).

CBC developed after prior tamoxifen was more often of the HER2-positive or triple negative-subtype, and had a significantly higher AIB1. This is in accordance with previously suggested endocrine treatment escape mechanisms such as loss of ER-dependence and/or activation of an alternative proliferative pathway such as HER2/neu. Indeed, the ER and HER2 signaling pathways interact on several levels, their co-expression is associated with relative endocrine therapy resistance, and dual targeting has demonstrated a clinical benefit [29].

These results are also in line with preclinical studies showing increased AIB1-expression in tamoxifen-resistant cell lines [16–18]. However, previous results regarding AIB1 in relation to endocrine treatment are conflicting. We have in an adjuvant randomized trial and in independent cohorts shown a high AIB1 to improve tamoxifen response [13, 14]. These results have also been confirmed in another randomized tamoxifen trial [15]. Conversely, high AIB1 has also been associated with endocrine treatment resistance [8, 9]. This may possibly be explained by differences in AIB1-function in different treatment settings. In the naïve setting a high AIB1 seems to correlate to improved tamoxifen response, possibly since it indicates a functional, active ER-signaling pathway which is effectively blocked by tamoxifen. However, overexpression of AIB1 in the resistant setting might instead indicate reactivation of ER-signaling, now irresponsive to tamoxifen. Another possibility is an interaction between AIB1 and other signaling pathways [30–33], inducing hormone-independent proliferation. Indeed, AIB1 has been speculated to be the limiting factor for several pathways (including the ER), deciding which one is activated [34, 35]. When one pathway is blocked, the other takes over.

In accordance with our previous studies in the adjuvant setting for a primary breast cancer, we found tamoxifen to improve survival in ER-positive AIB1-high CBC not previously exposed to the drug. Though ER-positive AIB1-high CBC developed despite prior tamoxifen had an equally bad prognosis regardless if tamoxifen was given or not. Consistent with previous studies, tamoxifen to patients with AIB1-low tumors did not further improve prognosis, regardless if this drug had been given or not before. However despite the large initial cohort, numbers were markedly reduced in subgroup analyses, reducing statistical power.

As in previous studies AIB1 correlated to a more aggressive tumor-phenotype (ER- and/or PR-negativity, HER2-overexpression, high Ki67, and a high TNM-stage) [7–12]. Previous studies have suggested a relationship between AIB1 and HER2 [8, 9, 36], although this has been investigated only in a few clinical trials [8, 9]. One reason for this may be that AIB1+HER2+ tumors are only a small subgroup of the total number of breast cancers. In the study by Osborne et al. there were 10 AIB1+HER2+ patients in the cohort with no adjuvant therapy (n = 119) and 25 in the tamoxifen treated cohort (n = 187), while Kirkegaard’s study of a tamoxifen treated cohort (n = 402) contained only 20 AIB1+HER2+ patients. In this study HER2 and AIB1 were each associated with a worse prognosis, regardless of concomitant expression of the other factor. However, as in previous studies, HER2-positive AIB1-positive/negative patients were few (Table 1), and conclusions possible to draw limited due to lack of statistical power.

Interestingly, the largest changes in AIB1, HER2, and Ki67-expression in relation to prior tamoxifen were observed in ER-negative CBC. This could be due to several treatment escape mechanisms being activated simultaneously. In addition, the lower Ki67 in ER-negative CBC after prior tamoxifen may reflect a gradual shift from an ER-positive phenotype with lower proliferation to a more aggressive ER-negative phenotype.

The present cohort represents, to our knowledge, the largest existing cohort with available tumor material from both breast tumors. Previous CBC-studies are instead either based on only register data or small patient cohorts. Register data is often incomplete, uncertain, and does not allow studies of new tumor markers such as AIB1. In regard to previous CBC-studies based on available tumor material, these include as few as 10–60 patients [37–46], with several even less than 15 patients [38, 39, 41, 43]. Hence, from a CBC-perspective, this is a unique and very large cohort. However, some potential sources of bias should still be considered. For example, even with a large initial cohort numbers are quickly reduced as subgroups are constructed in relation to treatment and tumor characteristics. Hence, subgroup analysis will include fewer events, reducing statistical power. In addition, analyses showed a higher percentage of AIB1-low tumors in the earlier calendar periods. Reasons may be increased use of adjuvant treatment over time, but also methodological changes in tumor fixation and/or antigen degradation in the oldest samples. However, the proportion of AIB1-high tumors remained relatively consistent, and analyses were adjusted for calendar period of diagnosis. Hence, this possible misclassification between medium and low AIB1 should not significantly affect our results. Finally, compared to previous studies this cohort included many tumors with medium AIB1-scores. Hence, we chose to analyze AIB1-expression in three stages instead of using one cut-off (<5 *vs*. ≥5). Reasons could again be older tumor-samples making evaluation more difficult. However, another possibility is a true higher expression in this unique cohort. CBC-patients likely have an increased breast cancer risk due to genetic and environmental factors, and this may affect tumor AIB1-expression. In addition, prior treatment may increase AIB1-levels of BC2.

In conclusion, CBC developed after prior tamoxifen show loss of ER-expression and activation of alternative proliferative pathways such as HER2/neu. This is consistent with resistance mechanisms previously suggested, and indicates CBC arising despite prior treatment to be a putative “*in vivo”*-model for studies of adjuvant treatment resistance. Previous studies regarding AIB1-expression in relation to tamoxifen have shown conflicting results. A high AIB1-expression has been correlated to both a good response to tamoxifen and tamoxifen resistance. As in previous studies we hereby found a high AIB1-expression to correlate to a worse prognosis in ER-positive breast cancer. Also in concordance with our previous studies in the adjuvant setting for primary breast cancer [13, 14], CBC with a high AIB1-expression not priory exposed to endocrine treatment seemed to respond well to tamoxifen. However, AIB1-high CBC developed after prior tamoxifen seemed to have an equally bad prognosis regardless if tamoxifen was given or not. In addition, previous tamoxifen was associated with increased CBC AIB1-expression, possibly reflecting a role of AIB1 in development of endocrine treatment resistance. These seemingly conflicting results in our study, as well as in previous studies, may possibly be explained by a different function of AIB1 in the treatment naïve and resistant setting. On explanation could be if a high AIB1 in the naïve setting indicates a functional, active ER-signaling pathway which is effectively blocked by tamoxifen. While overexpression of AIB1 in the resistant setting instead may indicate reactivation of ER-signaling, now irresponsive to tamoxifen. However, this theory needs further exploration in both preclinical and clinical studies. Patients receiving aromatase inhibitors were in this study few, but AIB1-expression in relation to response to these drugs is another very interesting question that warrants further investigation.

## Supporting Information

S1 Dataset Set used for analyses. (XLSX)

## References

[1] Anzick SL, Kononen J, Walker RL, Azorsa DO, Tanner MM, Guan XY, et al AIB1, a steroid receptor coactivator amplified in breast and ovarian cancer. Science. 1997;277(5328):965–8. . 9252329 10.1126/science.277.5328.965

[2] List HJ, Reiter R, Singh B, Wellstein A, Riegel AT. Expression of the nuclear coactivator AIB1 in normal and malignant breast tissue. Breast Cancer Res Treat. 2001;68(1):21–8. . 11678305 10.1023/a:1017910924390

[3] Bautista S, Valles H, Walker RL, Anzick S, Zeillinger R, Meltzer P, et al In breast cancer, amplification of the steroid receptor coactivator gene AIB1 is correlated with estrogen and progesterone receptor positivity. Clin Cancer Res. 1998;4(12):2925–9. . 9865902

[4] Murphy LC, Simon SL, Parkes A, Leygue E, Dotzlaw H, Snell L, et al Altered expression of estrogen receptor coregulators during human breast tumorigenesis. Cancer research. 2000;60(22):6266–71. . 11103781

[5] Azorsa DO, Cunliffe HE, Meltzer PS. Association of steroid receptor coactivator AIB1 with estrogen receptor-alpha in breast cancer cells. Breast Cancer Res Treat. 2001;70(2):89–101. . 11768608 10.1023/a:1012972808558

[6] Guan XY, Xu J, Anzick SL, Zhang H, Trent JM, Meltzer PS. Hybrid selection of transcribed sequences from microdissected DNA: isolation of genes within amplified region at 20q11-q13.2 in breast cancer. Cancer research. 1996;56(15):3446–50. . 8758910

[7] Dihge L, Bendahl PO, Grabau D, Isola J, Lovgren K, Ryden L, et al Epidermal growth factor receptor (EGFR) and the estrogen receptor modulator amplified in breast cancer (AIB1) for predicting clinical outcome after adjuvant tamoxifen in breast cancer. Breast Cancer Res Treat. 2008;109(2):255–62. . 17636398 10.1007/s10549-007-9645-1

[8] Kirkegaard T, McGlynn LM, Campbell FM, Muller S, Tovey SM, Dunne B, et al Amplified in breast cancer 1 in human epidermal growth factor receptor—positive tumors of tamoxifen-treated breast cancer patients. Clin Cancer Res. 2007;13(5):1405–11. . 17332282 10.1158/1078-0432.CCR-06-1933

[9] Osborne CK, Bardou V, Hopp TA, Chamness GC, Hilsenbeck SG, Fuqua SA, et al Role of the estrogen receptor coactivator AIB1 (SRC-3) and HER-2/neu in tamoxifen resistance in breast cancer. Journal of the National Cancer Institute. 2003;95(5):353–61. . 12618500 10.1093/jnci/95.5.353

[10] Bouras T, Southey MC, Venter DJ. Overexpression of the steroid receptor coactivator AIB1 in breast cancer correlates with the absence of estrogen and progesterone receptors and positivity for p53 and HER2/neu. Cancer research. 2001;61(3):903–7. . 11221879

[11] Bertelsen L, Bernstein L, Olsen JH, Mellemkjaer L, Haile RW, Lynch C, et al Effect of Systemic Adjuvant Treatment on Risk for Contralateral Breast Cancer in the Women´s Environment, Cancer and Radiation Epidemiology Study. J National Cancer Institute. 2008;100(1):32–40. 10.1093/jnci/djm267 18159070

[12] Hudelist G, Czerwenka K, Kubista E, Marton E, Pischinger K, Singer CF. Expression of sex steroid receptors and their co-factors in normal and malignant breast tissue: AIB1 is a carcinoma-specific co-activator. Breast Cancer Res Treat. 2003;78(2):193–204. . 12725419 10.1023/a:1022930710850

[13] Alkner S, Bendahl P, Grabau D, Malmstrom P, Ferno M, Ryden L, et al The role of AIB1 and PAX2 in primary breast cancer: validation of AIB1 as a negative prognostic factor. Ann Oncol. 2013;24(5):1244–52. Epub 2012/12/12. 10.1093/annonc/mds613 . 23230135

[14] Alkner S, Bendahl PO, Grabau D, Lovgren K, Stal O, Ryden L, et al AIB1 is a predictive factor for tamoxifen response in premenopausal women. Ann Oncol. 2010;21(2):238–44. Epub 2009/07/25. 10.1093/annonc/mdp293 . 19628566

[15] Weiner M, Skoog L, Fornander T, Nordenskjold B, Sgroi DC, Stal O. Oestrogen receptor co-activator AIB1 is a marker of tamoxifen benefit in postmenopausal breast cancer. Annals of Oncology. 2013;24(8):1994–9. 10.1093/annonc/mdt159 . 23670096 PMC3718507

[16] Su Q, Hu S, Gao H, Ma R, Yang Q, Pan Z, et al Role of AIB1 for tamoxifen resistance in estrogen receptor-positive breast cancer cells. Oncology. 2008;75(3–4):159–68. Epub 2008/10/02. doi: 000159267 [pii] 10.1159/000159267 . 18827493

[17] Zhao W, Zhang Q, Kang X, Jin S, Lou C. AIB1 is required for the acquisition of epithelial growth factor receptor-mediated tamoxifen resistance in breast cancer cells. Biochem Biophys Res Commun. 2009;380(3):699–704. Epub 2009/03/17. doi: S0006-291X(09)00215-0 [pii] 10.1016/j.bbrc.2009.01.155 . 19285025

[18] Redmond AM, Bane FT, Stafford AT, McIlroy M, Dillon MF, Crotty TB, et al Coassociation of estrogen receptor and p160 proteins predicts resistance to endocrine treatment; SRC-1 is an independent predictor of breast cancer recurrence. Clin Cancer Res. 2009;15(6):2098–106. Epub 2009/03/12. doi: 1078-0432.CCR-08-1649 [pii] 10.1158/1078-0432.CCR-08-1649 . 19276281

[19] Atanaskova N, Keshamouni VG, Krueger JS, Schwartz JA, Miller F, Reddy KB. MAP kinase/estrogen receptor cross-talk enhances estrogen-mediated signaling and tumor growth but does not confer tamoxifen resistance. Oncogene. 2002;21(25):4000–8. . 12037682 10.1038/sj.onc.1205506

[20] Torres-Arzayus MI, Yuan J, DellaGatta JL, Lane H, Kung AL, Brown M. Targeting the AIB1 oncogene through mammalian target of rapamycin inhibition in the mammary gland. Cancer research. 2006;66(23):11381–8. . 17145884 10.1158/0008-5472.CAN-06-2316

[21] Myers E, Hill AD, Kelly G, McDermott EW, O'Higgins NJ, Buggy Y, et al Associations and interactions between Ets-1 and Ets-2 and coregulatory proteins, SRC-1, AIB1, and NCoR in breast cancer. Clin Cancer Res. 2005;11(6):2111–22. . 15788656 10.1158/1078-0432.CCR-04-1192

[22] Sandberg ME, Hartman M, Klevebring D, Eloranta S, Ploner A, Hall P, et al Prognostic implications of estrogen receptor pattern of both tumors in contralateral breast cancer. Breast Cancer Res Treat. 2012 Epub 2012/05/25. 10.1007/s10549-012-2096-3 . 22622811

[23] Alkner S, Bendahl PO, Ferno M, Manjer J, Ryden L. Prediction of outcome after diagnosis of metachronous contralateral breast cancer. BMC Cancer. 2011;11:114 Epub 2011/04/01. 10.1186/1471-2407-11-114 21450091 PMC3080341

[24] Hartman M, Czene K, Reilly M, Adolfsson J, Bergh J, Adami HO, et al Incidence and prognosis of synchronous and metachronous bilateral breast cancer. J Clin Oncol. 2007;25(27):4210–6. . 17878475 10.1200/JCO.2006.10.5056

[25] Alkner S, Ehinger A, Bendahl PO, Ryden L, Ferno M. Prognosis, stage and oestrogen receptor status of contralateral breast cancer in relation to characteristics of the first tumour, prior endocrine treatment and radiotherapy. Eur J Cancer. 2015 10.1016/j.ejca.2015.07.016 . 26243193

[26] Song X, Zhang C, Zhao M, Chen H, Liu X, Chen J, et al Steroid Receptor Coactivator-3 (SRC-3/AIB1) as a Novel Therapeutic Target in Triple Negative Breast Cancer and Its Inhibition with a Phospho-Bufalin Prodrug. PloS one. 2015;10(10):e0140011 10.1371/journal.pone.0140011 26431029 PMC4592245

[27] Balmer NN, Richer JK, Spoelstra NS, Torkko KC, Lyle PL, Singh M. Steroid receptor coactivator AIB1 in endometrial carcinoma, hyperplasia and normal endometrium: Correlation with clinicopathologic parameters and biomarkers. Mod Pathol. 2006;19(12):1593–605. . 16980945 10.1038/modpathol.3800696

[28] Goldhirsch A, Winer EP, Coates AS, Gelber RD, Piccart-Gebhart M, Thurlimann B, et al Personalizing the treatment of women with early breast cancer: highlights of the St Gallen International Expert Consensus on the Primary Therapy of Early Breast Cancer 2013. Ann Oncol. 2013;24(9):2206–23. 10.1093/annonc/mdt303 23917950 PMC3755334

[29] Mehta A, Tripathy D. Co-targeting estrogen receptor and HER2 pathways in breast cancer. Breast (Edinburgh, Scotland). 2014;23(1):2–9. 10.1016/j.breast.2013.09.006 . 24176518

[30] Louie MC, Zou JX, Rabinovich A, Chen HW. ACTR/AIB1 functions as an E2F1 coactivator to promote breast cancer cell proliferation and antiestrogen resistance. Molecular and cellular biology. 2004;24(12):5157–71. . 15169882 10.1128/MCB.24.12.5157-5171.2004 PMC419858

[31] Torres-Arzayus MI, Font de Mora J, Yuan J, Vazquez F, Bronson R, Rue M, et al High tumor incidence and activation of the PI3K/AKT pathway in transgenic mice define AIB1 as an oncogene. Cancer cell. 2004;6(3):263–74. . 15380517 10.1016/j.ccr.2004.06.027

[32] Shou J, Massarweh S, Osborne CK, Wakeling AE, Ali S, Weiss H, et al Mechanisms of tamoxifen resistance: increased estrogen receptor-HER2/neu cross-talk in ER/HER2-positive breast cancer. Journal of the National Cancer Institute. 2004;96(12):926–35. . 15199112 10.1093/jnci/djh166

[33] Wang M, Zhao F, Li S, Chang AK, Jia Z, Chen Y, et al AIB1 cooperates with ERalpha to promote epithelial mesenchymal transition in breast cancer through SNAI1 activation. PloS one. 2013;8(6):e65556 10.1371/journal.pone.0065556 23762395 PMC3676316

[34] Torres-Arzayus MI, Zhao J, Bronson R, Brown M. Estrogen-dependent and estrogen-independent mechanisms contribute to AIB1-mediated tumor formation. Cancer research. 2010;70(10):4102–11. Epub 2010/05/06. 10.1158/0008-5472.CAN-09-4080 20442283 PMC2879596

[35] Hu ZZ, Kagan BL, Ariazi EA, Rosenthal DS, Zhang L, Li JV, et al Proteomic analysis of pathways involved in estrogen-induced growth and apoptosis of breast cancer cells. PloS one. 2011;6(6):e20410 10.1371/journal.pone.0020410 21738574 PMC3124472

[36] Hurtado A, Holmes KA, Geistlinger TR, Hutcheson IR, Nicholson RI, Brown M, et al Regulation of ERBB2 by oestrogen receptor-PAX2 determines response to tamoxifen. Nature. 2008;456(7222):663–6. Epub 2008/11/14. doi: nature07483 [pii] 10.1038/nature07483 19005469 PMC2920208

[37] Matsuo K, Fukutomi T, Tsuda H, Akashi-Tanaka S, Shimizu C, Hasegawa T. Differences in estrogen receptor status, HER2, and p53 comparing metachronous bilateral breast carcinoma. J Surg Oncol. 2001;77(1):31–4. . 11344480 10.1002/jso.1062

[38] Janschek E, Kandioler-Eckersberger D, Ludwig C, Kappel S, Wolf B, Taucher S, et al Contralateral breast cancer: molecular differentiation between metastasis and second primary cancer. Breast Cancer Res Treat. 2001;67(1):1–8. . 11518461 10.1023/a:1010661514306

[39] Tse GM, Kung FY, Chan AB, Law BK, Chang AR, Lo KW. Clonal analysis of bilateral mammary carcinomas by clinical evaluation and partial allelotyping. American journal of clinical pathology. 2003;120(2):168–74. . 12931545 10.1309/6YEP-MCHA-CPG2-BD15

[40] Bachleitner-Hofmann T, Pichler-Gebhard B, Rudas M, Gnant M, Taucher S, Kandioler D, et al Pattern of hormone receptor status of secondary contralateral breast cancers in patients receiving adjuvant tamoxifen. Clin Cancer Res. 2002;8(11):3427–32. . 12429630

[41] Seo MY, Rha SY, Yang SH, Kim SC, Lee GY, Park CH, et al The pattern of gene copy number changes in bilateral breast cancer surveyed by cDNA microarray-based comparative genomic hybridization. Int J Mol Med. 2004;13(1):17–24. . 14654965

[42] Teixeira MR, Ribeiro FR, Torres L, Pandis N, Andersen JA, Lothe RA, et al Assessment of clonal relationships in ipsilateral and bilateral multiple breast carcinomas by comparative genomic hybridisation and hierarchical clustering analysis. British journal of cancer. 2004;91(4):775–82. . 15266323 10.1038/sj.bjc.6602021 PMC2364777

[43] Banelli B, Casciano I, Di Vinci A, Gatteschi B, Levaggi A, Carli F, et al Pathological and molecular characteristics distinguishing contralateral metastatic from new primary breast cancer. Ann Oncol. 2010;21(6):1237–42. Epub 2009/10/31. 10.1093/annonc/mdp470 . 19875753

[44] Russnes HG, Kuligina E, Suspitsin EN, Voskresenskiy DA, Jordanova ES, Cornelisse CJ, et al Paired distribution of molecular subtypes in bilateral breast carcinomas. Cancer genetics. 2011;204(2):96–102. Epub 2011/04/21. 10.1016/j.cancergencyto.2010.09.012 . 21504707

[45] Suspitsin EN, Sokolenko AP, Togo AV, Lazareva YR, Turkevich EA, Matsko DE, et al Nonrandom distribution of oncogene amplifications in bilateral breast carcinomas: Possible role of host factors and survival bias. Int J Cancer. 2007;120(2):297–302. 10.1002/ijc.22265 . 17066426

[46] Kuligina E, Grigoriev MY, Suspitsin EN, Buslov KG, Zaitseva OA, Yatsuk OS, et al Microsatellite instability analysis of bilateral breast tumors suggests treatment-related origin of some contralateral malignancies. J Cancer Res Clin Oncol. 2007;133(1):57–64. 10.1007/s00432-006-0146-0 . 16900353 PMC12160774

